# Adopting international recommendations to design a model for maternal health service to cope with pandemic disruption for Indonesian primary care

**DOI:** 10.1186/s12884-023-05433-8

**Published:** 2023-03-01

**Authors:** Fitriana Murriya Ekawati, Mumtihana Muchlis, Amita Tuteja

**Affiliations:** 1 Department of Family and Community Medicine, Faculty of Medicine, Public Health and Nursing, Universitas Gadjah Mada, Jalan Farmako Sekip Utara Sleman, Yogyakarta, Indonesia; 2 Iwoimendaa Primary Health Center, Kolaka Regency, South-east Sulawesi, Indonesia; 3 Department of General Practice, University of Melbourne, Victoria, Australia

**Keywords:** Maternal health, Maternity care, Model, Pandemic, Indonesia, Low-and-middle income countries

## Abstract

**Background:**

Limited evidence is available as the reference for the model of care on providing maternity care in low-and-middle-income countries (LMICs) to cope with pandemic disruption. This study aimed to adopt international recommendations to develop the model of care with the context of Indonesian settings.

**Methods:**

Four codesign workshops and substitute interviews with stakeholders, covering the (i) exploration of service provision during the pandemic, (ii) adoption of international recommendations, (iii) designing and (iv) finalising model of care for maternal health services in primary care under the COVID-19 pandemic. The study took place in Yogyakarta Province Indonesia from July-November 2021. The participants were general practitioners, midwives, nurses, patients, and obstetricians. The data were analysed thematically.

**Results:**

Twenty-three participants were recruited. As many as 23, 16, 14 and 16 participants participated in the first to fourth codesign workshops or substitute interviews. Key recommendations agreed upon in the workshop were health screening, maintaining antenatal-postnatal breastfeeding care, limiting visitors, using telemedicine, and creating a multidisciplinary team to provide the care. A model of care for improving maternal service was also agreed and received suggestions from the participants. Identified barriers to the recommendation implementation, such as the available clinical resources and negotiating providers’ authority in practice.

**Conclusion:**

Recommendations and the model of care for improving maternity care in Indonesia are beneficial to be implemented in Indonesian primary care during the COVID-19 pandemic. Further research includes pilot studies to explore the acceptability of the model and recommendation implementation in practice.

**Supplementary Information:**

The online version contains supplementary material available at 10.1186/s12884-023-05433-8.

## Plain language summary

Much evidence on maternal care is available, however, little is known about their potential adoption for improving maternal health services in Indonesian primary care during the COVID-19 pandemic. This study reports the adoption of international recommendations to develop a model for improving maternal health services in primary care to cope with disruptions caused by the COVID-19 pandemic, including potential barriers of recommendation uptake in practice. Further research is desired to pilot the recommendation in practice.

**Supplementary Information:**

The online version contains supplementary material available at 10.1186/s12884-023-05433-8.

## Background

The COVID-19 pandemic has disrupted the way maternity care is provided across countries [1]. A modelling of maternal services has predicted that pandemic disruption would decrease the quality of maternal health. While the impact of the infection to pregnant women and the related indirect effects of the pandemic, such as service closures, may result in additional maternal and infant mortality in low-and-middle-income countries (LMICs) such as in Indonesia [2, 3].

In Indonesia, primary maternal care is the most convenient health care service for patients and is accessed by more than 90% of pregnant women [4]. However, the services were challenged for their capacity to meet the patients' demand due to its limited human and clinical resources. The backbone of primary health care clinic in Indonesia, Puskesmas (Pusat Kesehatan Masyarakat/Community Health Center) for example, had to reduce to half their standard capacity, particularly if one of the staff tested positive for COVID-19. The staff and close contacts had to isolate, and the centre was closed for deep cleaning. While at the same time, many private practices chose to close their practice due to their inability to implement COVID-19 safety measures [5–8].

Routine maternal care in Indonesia has also been re-arranged to respond to the spread of COVID-19 infection and to maintain necessary access for the patients [1]. For example, telehealth visits were encouraged wherever possible, and decreased number for antenatal care visits and face-to-face maternity class to limit spread of the disease. However, with these extensive changes and challenges of service, little evidence reports these detailed situation in Indonesia, even when the country was the epicentre of COVID-19 Pandemic in July–August 2021 [9]. Previous research about maternity care in Indonesia in the early pandemic reported the overwhelming situations and frustration of the providers due to lack of personal protective equipment (PPE) and limited guidance when providing care for pregnant women [10]. They also mentioned the reduced community services in response to restriction during the COVID-19 pandemic without further alternative to care for the women [11].

Several guidelines for maternal health have been issued by the Indonesian Ministry of Health to facilitate the functioning of primary care in COVID-19 pandemic. These included guidelines for Puskesmas service [2] and guidelines for pregnant women, maternity, postpartum, and newborn: 1^st^ [12] and the 2^nd^ revision [13]. These general guidelines emphasized on social distancing, wearing masks and washing hands at Puskesmas for the staff and patients, as well as telemedicine and health consultations. However, limited detailed evidence-based recommendation on maternal health service is available in those guidelines, such as detailed procedures and explanations, models of care for women, maternal health examinations and monitoring. There was also limited information on prereferral procedures to and after hospitalization [10], which therefore a detailed reference on the model of care to prepare the provider to adapt maternity services to COVID 19 was needed.

While a volume of evidence in maternal care during the pandemic is available in the global literature [14–16], there is limited translation-into-practice of those evidence available for LMICs as such in Indonesia. Some of the available evidence of the model of maternity care that likely available in LMICs are from the World Health Organization (WHO) and a publication from Kenya [17, 18], stating the importance of continuing maternal care service for women during pandemic. The knowledge gaps compared to the global evidence then may led to challenges in services provided through primary care. For instance, during the transitioning to telemedicine or services following social distancing norms, many patients did not have sufficient means of technology to use the service or were unable to operate the telemedicine system. Some of them were also not familiar or comfortable with communication technology [19, 20].

This research aims to fill the gaps of provision of maternal health services during this pandemic by adopting international recommendations for improving maternal health service and developing a model of care suitable for the context of maternal health in Indonesian primary care. The recommendation and developed model will cover essential care guidance and approaches required during the pandemic to maintain maternity services, added with consideration from the primary care stakeholders. Additionally, it will include supporting arrangement for primary care collaboration with secondary and tertiary care required for the context of maternity care in Indonesia.

## Methods

### Design

**Fig. 1:**
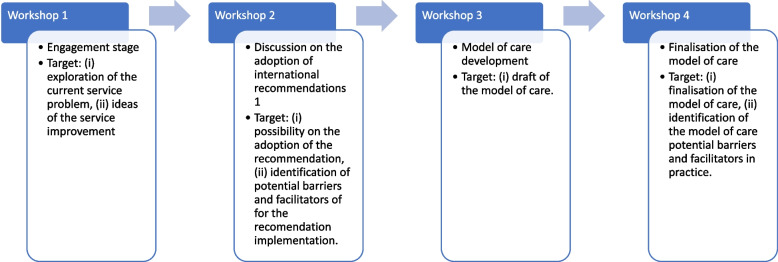
Workflow of the workshops

We applied participatory research techniques and codesign principles to guide our study. Codesign is a process of designing and developing innovation by involving all users and stakeholders involved in the innovation [21, 22]. In codesign process, all participants actively participate and provide opinions to design the innovation, while the role of the researcher is to facilitate the discussion to achieve innovation. More specifically, our study adopted an experienced-based codesign approach [23, 24] by involving all stakeholders experienced in providing care for pregnant women in primary care. Their vast experience in maternity care during the COVID-19 pandemic situation were beneficial to provide judgement or rationales on the adoption of the recommendations identified from our literature review, and to develop a model for the maternity care. Therefore, the expected model could be more tailored and suitable to the needs of the primary care providers [25, 26]. To allow a more dynamic discussion between the participants, this study applied qualitative focus groups methods for its data collection, that are covered in the designated workshops below [26, 27], and the detailed workflow of the design and targets of each workshop are provided in Fig. 1:

1. Workshop 1-aimed to engage the stakeholders with the research context. It mainly explored problems and participants' expectations of maternity care during the COVID-19 pandemic.
2. Workshop 2-aimed to seek consensus on the adoption of maternal care recommendations identified in our literature review, and their potential barriers and facilitators in practice.
3. Workshop 3-aimed to develop the model of maternity care during the COVID-19 pandemic
4. Workshop 4-aimed to finalise the recommendation adoption and the pandemic specific maternal health service model.

### Settings

The setting of this research was in Yogyakarta province, Indonesia. The province consists of five districts: City, Sleman, Bantul, Gunung Kidul, and Kulon Progo. The maternal health management in the province was similar across the regencies i.e., through Puskesmas and hospitals, with a few minor differences in each district. For instance, Bantul has a dedicated COVID-19 hospital from 2020–2021. On August 2021, there was 1,498 infected pregnant women and 67 deaths in Yogyakarta [28, 29]. There were also 27 referral hospitals to tackle the pandemic; however, the bed occupancy rate (BOR) reached 100% during the peak of Delta outbreak, and the tertiary hospital in the province collapsed due to its incapability to manage more patients [30].

### Participants and recruitment

We included all stakeholder groups including health workers and patients. They were general practitioners (GPs), midwives, nurses, and obstetricians, who were involved in the care of pregnant women, and patients who received care during the pandemic. The patient participants are those GPs’ or midwives’ patients who visited the canters during the pandemic. The inclusion criteria to participate in this study were (i) being a health worker (such as midwives, GPs, or nurses) with a minimum of two years of practice experience and (ii) understanding of the context of primary care practice in Indonesia. Recruitment of health-worker participants was conducted through purposive maximum variation sample design to represent the five districts in Yogyakarta and representativeness of private-and public practice. The participants were identified from the authors’ professional networks, not limited to certain health centers, and were invited through professional means of communication such as by messages, email, WhatsApp Group or by phone invitation. Patient participants of the study were recruited through their GPs and midwives participating in the research. The prospective participants who were interested in participating in the study were then further approached by the research team and explained about the study, including the required completion of the consent form.

### Review of the literature to identify improvements for Indonesian maternal care

Before the study, two authors with their team conducted a literature review identifying potential recommendations for improving maternal health services in Indonesia during the pandemic situation [31]. Results of the review identified four broad recommendations which had potential for application to the maternity care in Indonesian primary care settings. They include recommendations from a) Breastfeeding guidelines in the context of COVID-19 by Centers for Disease Control and Prevention (CDC [32], b) Perinatal and Neonatal COVID-19 Management of Federation of Obstetrics and Gynaecology Society of India (Chawla, Chirlaet.al. [33], c) COVID-19 Technical Brief for Maternity services (United Nations Population Fund (UNFPA [34], and d) Clinical care of pregnant and postpartum women with COVID‐19: Living recommendations from the National COVID‐19 Clinical Evidence Taskforce (Vogel, Tendalet.al. [35]. The identified recommendations are enclosed in Supplementary file 1.

After careful analysis and appraisal of those recommendations, It became obvious that the Indonesian guidelines only covered general advice such as the use of PPE, social distancing, and the referral to hospitals [12, 13] and there was a clear paucity on how to adapt these guidelines to maternity settings in primary care. Our review identified this gap and enumerated points relevant for improvements for clinical and organisational care in primary care service for women and newborn children. This included detailed procedures regarding triage, care for women during ante to postnatal care, detailed procedures on breastfeeding and well-baby visits in primary care, which were not or only stated superficially in the Indonesian guidelines. Of note, our review also identified that the essence of team approach for maternal health, that was not covered in the Indonesian guidelines, while this consideration is important for providing care during the COVID-19 pandemic. The recommendations (Supplementary file 1) then were brought and presented to the participants to achieve their consensus for their suitability for Indonesian practice at Workshop 2.

### Data collection

The recruited participants were asked to participate in the workshop 1–4, or substitute interviews. The workshops were conducted between July to November 2021 online through Zoom teleconference https://zoom.us. The participants attending the focus group workshops were divided into several rooms based on their professional group. This procedure was strictly followed during Workshop 1, and 2 to anticipate hierarchical barriers between the participants from different professional backgrounds [36, 37]. While during Workshop 3 and 4, all the participants attended the same session with no breakout rooms. Participants who could not attend the meeting were followed-up with an interview with similar guiding questions as the FG workshop (Table 1). All of the guiding questions have previously been tested in the simulation workshop before their actual use in the study.

**Table 1:** Guiding questions for codesign workshops

Workshop number	Guiding questions for participants
Workshop 1	1. Opening: the moderator leads the discussion and provides an explanation of this research and informed consent 2. Moderation of discussion 1 (opening and exploration): research participants will be asked the following questions: - What is your experience of providing maternal services in primary care during this pandemic? - What things went well in primary care? - What things need improvement? - What are your expectation for providing maternal services in primary care?
Workshop 2,3	Moderation of discussion 2,3 (codesign): research participants will be asked to analyze the list of maternal service recommendations obtained from the analysis of the latest scientific evidence on maternal management. They were asked: - In your opinion, how are these maternal recommendations? - Can the procedures be adopted in Indonesia? - What are some of the barriers and supports for the procedure?
Workshop 4	Moderation of discussion 4 (model finalization): before the 4th meetings, the researcher will draft a design for maternal care in primary care, participants will be asked to help finalize the design. They were asked: - In your opinion, how is the design of this maternal service model? - What are your further suggestions on this design? What are they? - What are the barriers and facilitators for implementing this model?

The workshops and interviews were conducted and moderated by the first and second authors, who are a female GP and a midwife in the province, with extensive experience of providing service and research in maternity care in Indonesia and are familiar to half of the participants. Experiences and suggestions from participants were discussed and summarized at each meeting, including the number of participants who agreed or disagreed with the proposed maternal procedure and design. These results were also presented at the following workshops for participants to validate or provide further feedback. At the Workshop 4, the authors drafted the maternal care model for the pandemic situation and presented it for participants’ feedback and approval.

### Data analysis

We used the thematic analysis technique for analysing the data. The analysis was conducted inductively and described as follows: (i) All the workshop recordings and substitute interviews were transcribed. (ii) The transcripts were then reviewed to identify meaningful quotes or suggestions from the participants, and in the second read the first author coded all the transcripts with the aid of NVIVO-12 software [38]. (iii) The second and third author then read the transcripts (third read), independently verified the coding tree and (iv) the significant and most representative quotes were grouped to elicit themes. (iv) Coding and data collection occurred concurrently and data saturation was noticed after the fourth workshop. (vi) Lastly, minor discrepancies in coding were resolved by series of discussion until consensus on thematic analysis was achieved, and the themes were then grouped to develop overarching themes [39]. In this paper, we presented relevant findings and the themes that contributed to the development of the model of maternity care in COVID-19 pandemic from each workshop.

### Rigour and trustworthiness of the study

We took multiple steps to ensure that the study findings are valid and transferable to clinical and research settings. The first and second author explicitly documented the progression of research and created an audit trail for future collaborators. The uniformity of the data collection was ensured by asking the participants with same questions in the same order during the workshops or their subsequent interviews. Our workshops and interviews were also always using open ended questions, including our prompts in the email and the WhatsApp groups to allow views from the participants. We also noted any non-verbal intonation or gestures from the participants during the workshops/interviews [40–42], and preserved authenticity by purposefully selecting participants and allowing them to freely articulate their needs. To achieve the participants’ validation, we circulated the summary and notes of each workshop using email and WhatsApp group communication. This helped to preserve criticality and obtain feedback about our proposed model.

The participants number in this study was limited, however the data saturation was ensured by reminding the participants to provide any suggestion or feedback regarding the results of the previous workshops until no more feedback was added, and that there was no more significant suggestions or views after the fourth workshop [43]. Rigour of the data analysis was also conducted by series of weekly meetings between the authors to validate the coding process until consensus on the data analysis and coding were reached. During this process, we also deliberated on our dual roles of clinicians and researchers and acknowledged our biases [36, 44, 45]. Reporting of the results of the study follows the COnsolidated criteria for REporting Qualitative research (COREQ) checklist (Supplementary file 2) to ensure a transparent reporting and credibility of the study [41, 46].

## Results

### Participants demography, and the dynamic of the workshops

Twenty-three participants were recruited for this study. Most were female (*n* = 22), midwives (*n* = 10), practiced in Puskesmas (*n* = 13) and had practice experiences for over 20 years (*n* = 14). The summary of participants' demographic profiles is presented in Table 2 and details of each participant are shown in Table 3. Workshop 1 consisted of 2 FGs, one FG for GPs (*n* = 2) and midwives (*n* = 10) each, and 11 interviews, including 3 interviews with patients. Workshop 2 also consisted of 2 FGs, with GPs (*n* = 2), midwives (*n* = 8), and six interviews. Workshop 3 consisted of 1 FG (GPs (n-3), nurse (*n* = 1), midwives (*n* = 7)), and three interviews, and the final workshop consisted of 1 FG (GPs (*n* = 2), nurses [1], midwives (*n* = 9)) and four interviews.

**Table 2:** Summary of participants characteristic

Category	*N* = 23
Sex	
a. Male	1
b. Female	22
Occupation	
a. General physician	4
b. Obstetrician	4
c. Midwife	10
d. Nurse	2
e. Patient	3
Level of Education	
a. Secondary school	1
b. Diploma	6
c. Bachelor	8
d. Master/ specialist	7
e. Doctoral	1
Workplace	
a. Puskesmas (Public Primary Care Clinic)	13
b. Hospital	4
c. Private midwife practice	3
d. Other (patients)	3
Practice experience	
a. 0–5 years	0
b. 6–10 years	4
c. 11–15 years	1
d. 16–20 years	1
e. > 20 years	14
f. n/a (patients)	3

**Table 3:** Detailed of the participants’ characteristic

Participant number	Gender	Occupation	Education	Workplace	Working experience	Workshop 1			Workshop 2			Workshop 3		Workshop 4	
						Substitute Interview	FG 1 (midwives)	FG 2 (GPs)	Substitute Interview	FG 1 (midwives)	FG 2 (GPs)	Substitute Interview	FG	Substitute interview	FG
**1**	Female	Nurse	Bachelor	Puskesmas	16–20 years	√	-	-	-	-	-	√	-	√	√
**2**	Female	Midwife	Bachelor	Puskesmas	> 20 years	-	√	-	-	√	-	-	√	-	√
**3**	Female	Midwife	Bachelor	Private midwife practice	> 20 years	-	√	-	-	√	-	-	√	-	√
**4**	Female	Patient	Bachelor	Other	n/a	√	-	-	-	√	-	-	-	√	-
**5**	Female	Patient	High school	Other	n/a	√	-	-	-	-	-	-	-	√	-
**6**	Male	Doctor	Master	Puskesmas	> 20 years	-	-	√	-	-	√	-	√	-	√
**7**	Female	Midwife	Diploma	Puskesmas	> 20 years	-	√	-	-	-	-	-	√	-	√
**8**	Female	Midwife	Bachelor	Puskesmas	6–10 years	-	√	-	-	√	-	-	√	-	√
**9**	Female	Midwife	Diploma	Puskesmas	6–10 years	-	√	-	-	-	-	-	-	-	√
**10**	Female	Patient	Diploma	Other	n/a	√	-	-	-	-	-	-	√	-	-
**11**	Female	Doctor	Bachelor	Puskesmas	6–10 years	-	-	√	-	-	√	-	√	-	√
**12**	Female	Midwife	Bachelor	Private midwife practice	> 20 years	-	√	-	-	√	-	-	-	-	√
**13**	Female	Midwife	Diploma	Puskesmas	> 20 years	-	√	-	-	-	-	-	-	-	-
**14**	Female	Midwife	Diploma	Puskesmas	> 20 years	-	√	-	-	√	-	-	√	-	√
**15**	Female	Midwife	Bachelor	Puskesmas	> 20 years	-	√	-	-	√	-	-	√	-	√
**16**	Female	Midwife	Master	Private midwife practice	> 20 years	√	-	-	-	√	-	-	√	-	√
**17**	Female	Doctor	Master	Puskesmas	11–15 years	√	-	-	√	-	-	√	-	-	-
**18**	Female	Obstetrician	Specialist	Hospital	> 20 years	√	-	-	√	-	-	-	-	-	-
**19**	Female	Obstetrician	Specialist	Hospital	> 20 years	√	-	-	√	-	-	-	-	-	-
**20**	Female	Nurse	Diploma	Puskesmas	6–10 years	√	-	-	√	-	-	-	-	-	-
**21**	Female	Doctor	Master	Puskesmas	> 20 years	√	-	-	√	-	-	-	-	√	-
**22**	Female	Obstetrician	Specialist	Hospital	> 20 years	√	-	-	√	-	-	√	-	-	-
**23**	Female	Obstetrician	Specialist	Hospital	> 20 years	√	-	-	-	-	-	-	-	-	-

The workshops lasted for one to three hours, and the interviews lengths were 15 to 45 min with adequate dynamic of the participant discussion. Participants who preferred to participate in the interviews were also able to express and elaborate most of their answers. The participants who did not participate in the workshops/interviews were not required to provide their reason for participation.

### Evaluation of the current maternal health services in primary care during the COVID-19 pandemic

#### Applied COVID-19 safety measures

All participants highlighted that in the Indonesian context maternity health services at primary care levels primarily dealt with patient screening using a questionnaire or short interviews related to their possible COVID-19 symptoms and close contacts. Patients were advised to use facemasks and to wash their hands using sanitizer or soap when visiting the clinics. Personal protective measurements (PPE), even though limited, were also available in practice and ready for the health worker's use, as mentioned by a participant below:

“We applied distancing measures and proper screening for women and all visitors in primary care, however, in the early (pandemic) phase, the major challenges were the lack of standardized personal protective equipment (PPE) and facilities. Primary health care, particularly private midwifery services, mainly focus on and is mandated to provide promotive and preventive services, managing the normal condition and are not set up to tackle the increase demand of infectious diseases outbreak. So, we were not well prepared. Late and unclear guides worsened it. Most of the guides indicated patients then have to refer to the hospital, and very little procedure is prescribed for us in primary care” (Workshop 1, FG 1, Participant 9).

#### Barriers of current care

Lacking practice guidance and authority were the main challenges of practicing maternity care during the COVID-19 pandemic in Indonesia. In the early phase of the pandemic, all COVID-19 patients were referred to hospitals. The primary care task was merely on screening and assisting with COVID-19 surveillance with limited guidance on the management, of patients including pregnant womenealth workers conducted minimal procedures for patient monitoring, and many pregnant women who deteriorated quickly could not be saved.

”The challenge of lacking guidance was experienced significantly during the Delta outbreak, where patients were stratified and those without symptoms isolated at home with telemonitoring from Puskesmas. Unfortunately, pregnant women with COVID-19 easily fall into deteriorated conditions, and with the lack of guidance for preventive measurements and care before referral, many could not be saved during their care at the hospital” (Substitute interview 1 for workshop 1, Participant 18).

Providing routine maternity care for women in primary care was also challenged by silos of authority and limited clinical resources. In planned labour events, women were asked to have a negative antigen/PCR (polymerase chain reaction) test, which at that time were not covered by the Indonesian JKN insurance (Jaminan Kesehatan Nasional/English: Indonesian National Insurance). In the event of emergency situations or natural labour events, not having a negative report was problematic in deciding the place of care and hospital admissions, while the midwives were not authorized to perform antigen tests in the first year of the pandemic as they could not claim it to the insurance. Besides that, during the peak of the Delta outbreak, many health providers isolated themselves and were not available to care for patients. The clinic management was further challenged by limited guideline available in practice for maternity services.

“Women in 3^rd^ trimester were directed to antigen test in Puskesmas and were retested again close to the labour. However, if they delivered in private practice, the test became problematic because midwives were seen as having no competency to do the test, even in emergency labour. Besides, the test was not covered by JKN insurance if it is conducted in our practice and not in Puskesmas” (Workshop 1, FG 1, Participant 15).

### Adoption of international recommendations

#### Practice needs responded

We summarized the needs of participants after Workshop 1, and it became obvious that detailed maternity specific guidance is needed for primary care settings in the COVID-19 pandemic. Therefore, Workshop 2 focused on the consensus of the adoption of the recommendations to Indonesian settings by appraising the recommendations for their suitability for implementation in Indonesian primary care practice. The recommendations were welcome, received comments and suggestions from the participants. Feedback from the participants particularly was on preparing referrals of pregnant women with COVID-19 complications to Indonesian PONEK (Pelayananan Obstetri Neonatus Emergency Komprehensif/ English: Comprehensive maternity and neonatal hospital/CEmONC) hospitals [47]. During the workshop, the participants also provide the roles of community resources, particularly, about conditions that were not covered by the JKN insurance, and their roles to help maintaining the number of antenatal care visits in line with national recommendations as well as referrals to the hospitals. These participants’ suggestions were then accommodated in the development of the model of care in Workshop 3 and 4 as below:

“There was a missing referral manual (Indonesian: manual rujukan) for COVID-19 as primary regulation for all health services levels, as well as no mapping COVD-19 referral hospital, for instance, hospital A for Puskesmas/private practice in areas A to C, etc. So, we struggle to look for available hospitals, which requires time and effort. It may be different if the district has a specific hospital for COVID-19 as well as community supports or from non-government organization as such we have in in Bantul” (Workshop 2, Participant 8)

“These complete recommendations are useful for their implementation in practice, however, the referral system was the problem from the initial phase to the second wave of the pandemic (Delta outbreak). We found it was very difficult to refer patients because hospitals reject patients for various reasons such as over-capacity, lack of facilities and financing, these should be anticipated in our model of care” (Substitute interview for Workshop 2, Participant 21).

#### Potential implementation barriers

According to the participants, potential barriers to the recommendation implementation in practice include negotiating practice authority with higher centres, the providers' knowledge of referral pathways and the patient’s literacy level. Many participants were still accustomed to their usual practice of referring women to the hospital immediately after pregnancy complications occurred. However, they also acknowledged that referral centres were busier than usual, and transfer of care would require long discussions and refusals, which therefore they needed to understand the referral preparation to anticipate this process. The participants also added, even though procedures were partially listed in the current Indonesian guidelines, GPs and midwives admitted that not all workers applied the recommendation in practice due to minimal rewards and their limited understanding. Therefore, they provided suggestions to accelerate the recommendation uptake, such as by providing endorsing policies and incentives to stimulate the providers' changing behaviours in practice.

“It would need more effort if Puskesmas has to handle COVID-19 patients because of limited facilities and health provider capacity. We also lacked proper incentive in primary health care, so it is not comparable with the risk. Therefore, there is a need to support health workers for the implementation, probably provide them with incentives or reward to apply them in practice appropriately” (Workshop 3, FG, Participant 6).

The participants also expressed the need to also elaborate the patients and educating them about the procedures. They mentioned that the women's literacy might also challenge the recommendation implementation as many women have a limited understanding of COVID-19 and how to prevent the infection in their family.

“Each patient’s literacy and perception are different. Some women can properly understand and follow midwives’ advice, but some must be taught regularly or have to be convinced first about COVID-19 risks because they do not believe it. (Once they understand) we can later apply the procedures optimally” (Workshop 3, FG, Participant 3).

### Model of maternal health service in primary care

At the end of the fourth workshop, the model of maternal health service in primary care achieved agreement from the participants. The model consists of three main domains of care settings namely a) community, b) primary care and c) hospitals. Finally, it incorporates supporting elements needed to collaborate between these three settings.

**Fig. 2:**
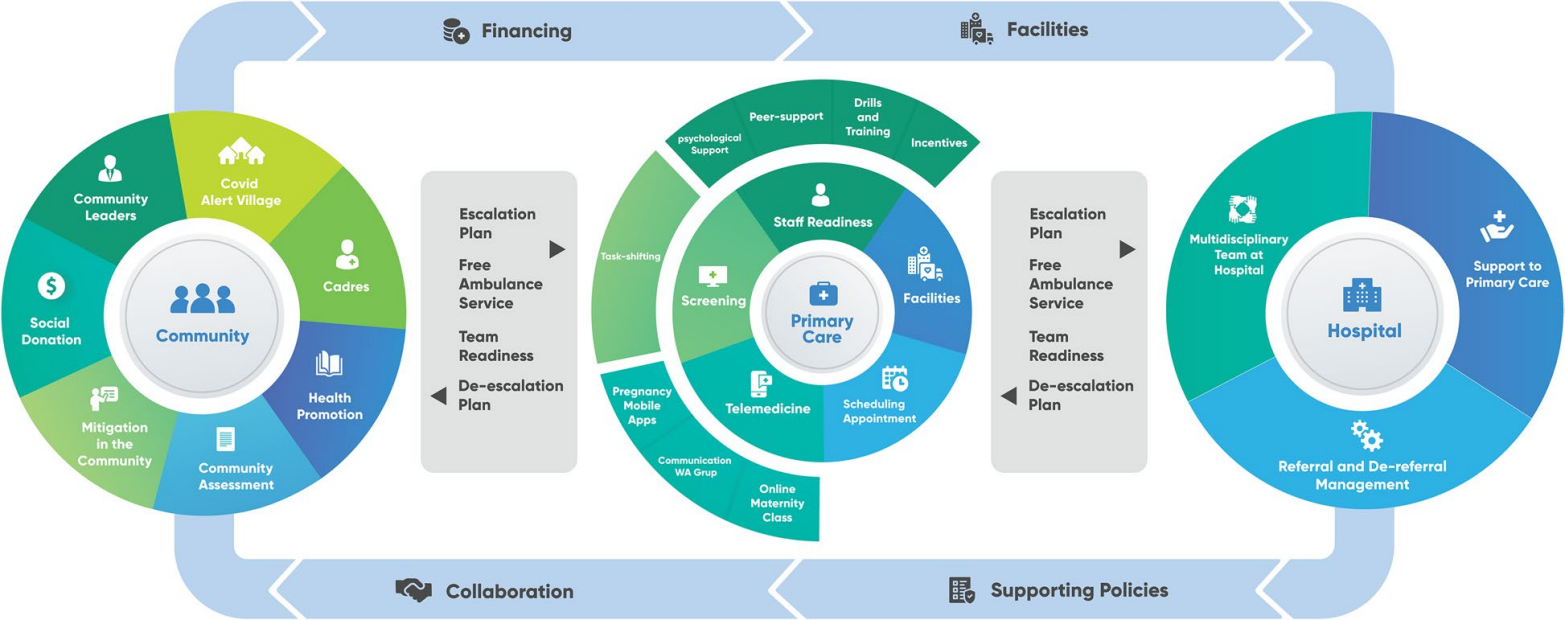
The model of maternal service agreed in the final workshop

In the community, maternal care for women includes community involvement to support pregnant women in the pandemic, how the community can help mitigate transmission in case pregnant women become infected and then encourage them to present to primary care service. At primary care level, the maternal services focus on providing continued safe antenatal care, the need of developing telemedicine, team preparation for outbreaks and screening of asymptomatic patients to prevent the spread of infection. The model also includes necessary escalation plan to accelerate referrals to the hospital with appropriate procedures before the referral, while hospitals also needs to inform primary care providers of the care given to the patients to increase collaborative care. Above all, the participants restated that to enable collaborations between these settings, adequate financial support and appropriate policies are needed. The model also reaffirms that primary care providers shall receive appropriate incentives for their service (Fig. 2).

“Communication and collaboration are keys in the referral system. We need adequate collaboration between Puskesmas or private midwives practice, and hospital; and it must be supported by health department by providing mechanism, incentives, endorsement policy and media communication. Then we also need to regularly disseminate, monitor and evaluate the service” (Workshop 4, FG, Participant 12).

“Community, such as health cadres, village workers, youth and women group should be involved because several patients are in self-isolation and they can support them in monitoring and providing meals, transportation and donation” (Substitute interview for Workshop 4, Participant 21).

## Discussion

Our study has adopted international recommendations for their readiness to be implemented in Indonesian settings and designed a model to guide the provision of maternal service in primary care settings. The recommendations were based on our systematic review [31], and have received suggestions from stakeholders involved in the study. Our generic model covers the main aspects of maternal care in Indonesia, as such in the community, primary care level and collaboration with the hospital. These levels are linked with an appropriate patient escalation-de-escalation plan, endorsing policy, financing and reward for the providers. The vital roles of the community were also highlighted that maternity care cannot be separated from the community due to a lack of resources in health care settings. The engagement between primary care providers and the community also reflects the spirit of ‘gotong-royong’ (mutual cooperation) between all of the elements in the community, that is essential to reduce the gap of resources in primary care settings. In addition, the endorsing policies will strengthen the uptake of the recommendations, including patients’ obedience to the recommendations [36, 37].

Our model is one of a few models of maternal health care developed in LMICs to assist providers in providing care for women during the pandemic. Some comparable models similar to ours was developed in Kenya, and the WHO recommendations for maintaining maternal service during the COVID-19 pandemic [17, 18]. Both of these models highlight the increasing roles of primary care and community midwives to provide quality care to women despite of the limited resources in health care provision. Both of them also recommend practical clinical and referral pathways including transportation arrangements from the community to primary and secondary care [35, 48]. We have therefore adopted these essentials in our model, and include them as the core elements for the service in micro primary care level (Supplementary file 2).

Our model can be directly implemented in Indonesian primary care settings. This will serve as a blueprint for health care providers to create local protocols for maternity service during the COVID-19 pandemic. Undoubtedly the success of the model of care and changes in maternity services in COVID-19 depends on a sustainable supply of clinical resources, as well as collaborative care between primary care and hospitals with endorsement on the primary care authority on providing essential maternity services. Finally, improving COVID-19 literacy among pregnant patients and their families will also lead to long-term improvement in maternal health.

Negotiating authority between providers in Indonesia is classic due to the hierarchical culture in the medical society [36, 37, 44]. Examination procedures in Indonesia were often attached to certain professionals rather than considering the essence of the examination itself. Even though this hierarchical barrier during the pandemic is less prominent [36], the sense of this power culture still occurs between the providers. In this case, for instance, PCR antigen swabs were previously performed exclusively by the pathologists or those working in the laboratory rather than conducted by any health professionals involved in the patient care [36, 49]. However, this practice has changed, allowing private midwives to test their patients before labour [50].

Despite its ability to showcase essential appropriate practices and elements of maternal care to cope with the COVID-19 pandemic, limitations of our codesign workshops include the small sample size and the restricted time to conduct the study. The sample of the study involved groups of stakeholders in maternal health services in Indonesia. However, as the study was conducted during the peak of the Delta outbreak in Indonesia (2021), not many providers had opportunities to participate in the study. The workshops were also provided through teleconference, potentially limiting the group discussion dynamic [51, 52]. Our review of literature on recommendations was also conducted before recommendations for vaccinating pregnant women were released. However, this study acknowledges and support the importance of COVID-19 vaccination in pregnant women [53, 54].

Results of the study can also be directly implemented to guide practice or policy in maternal health in other settings with similar practice environments. Nonetheless, continuous improvement in the model of maternal health services and care for women are needed is all countries, with attention to LMICs as the majority of the maternal mortality occurs in this setting. Even though the provision of the COVID-19 safety measures might have been released along with the increasing vaccination rates, challenges in Indonesia reminds on the equity of vaccine distribution and the low uptake of the booster doses [55, 56]. These may lead to the continuity of infection spread, and mutation of the virus itself may still occur. Therefore, health care providers must remain vigilant, apply safety measures and ensure the quality of the provided service. Finally, for further research is desired to pilot the model and recommendation in practice and evaluate the implementation before its extensive adoption in Indonesian settings. Successful implementation outcome measures would include numbers of patients receiving procedures according to the model or recommendation, utility of triage tools, quality of referrals, ease of referrals and feedback from the primary care workers involved in implementation.

## Conclusion

This study has provided consensus on adopting international recommendations and developed a model for improving maternity services during COVID-19 pandemic in Indonesian primary care. Collaborations developed during the peak of pandemic have to be ensured to maintain coordinated care between primary care practitioners and hospital society. Finally, codesign approaches of involving all stakeholders to design innovative solutions for local contexts have widespread applications in other LMICs. Further research is desired to pilot the uptake of the recommendations and the model in primary care practice.

## Supplementary Information

**Additional file 1.**

**Additional file 2.**

## Data Availability

Raw data of this manuscript are available from the corresponding author on reasonable request. The essential data regarding the results and supplements have been attached in the main text and supplementary files.
