# Supplementary material for: Adopting international recommendations to design a model for maternal health service to cope with pandemic disruption for Indonesian primary care
Source: BMC Pregnancy Childbirth. 2023 Mar 1;23:132. doi: 10.1186/s12884-023-05433-8 (PMC9975861; doi:10.1186/s12884-023-05433-8)
Supplement: Supplementary file 1 — Additional file 1. [file 12884_2023_5433_MOESM1_ESM.docx]

**Supplementary file 2.**

**IDENTIFIED RECOMMENDATIONS FOR IMPROVING MATERNAL HEALTH SERVICE IN iNDONESIAN PRIMARY CARE UNDER THE COVID-19 PANDEMIC**

| TOPIC | SUBTOPIC | RECOMMENDATIONS |
| --- | --- | --- |
| Triage | General principles | 1. Triage and screening for any COVID-19 symptoms and close contact of COVID-19 patients needs to be conducted for all women and their accompanying person who come to health facilities. 2. Anyone who reports fever and/or respiratory symptoms, needs to be considered as likely to have COVID-19. 3. Pregnant women living in high-density communities, and urban slums can be considered for their higher risks of contracting COVID-19 due to the high incidence of infectious diseases, overcrowded housing, and malnutrition. 4. Health workers should be vigilant that pregnant women with COVID-19 may experience mild symptoms (such as fatigue and headaches) and present with symptoms similar to other diseases e.g., malaria, dengue fever, and preeclampsia. 5. If health workers suspect a woman to have COVID-19 either due to COVID19 symptoms, the woman should be referred for further examination. |
|  | Triage in Ambulances / Transport | 1. Pregnant women suspected or confirmed with COVID-19 coming to emergency service should follow COVID-19 protocol. 2. If they need an ambulance, the women must notify the worker that they are in self-isolation due to suspected or confirmed COVID-19. 3. Before entering the hospital, women with COVID-19 must inform staff about their arrival (by phone). 4. When entering the hospital environment, women with COVID-19 are required to wear masks. 5. Pregnant women with COVID-19 should come to the hospital on their own if possible using a private vehicle. 6. If using public transport, the women must inform the driver of her condition. |
| Referral of pregnant women dengan COVID | Referring women with COVID | 1. Referral mechanisms for the procurement of emergency transportation can be coducted from primary care clinics to PONEK (Comprehensive maternity and neonatal hospital/CEmONC), then to the higher care if necessary. 2. Health workers should ensure that the mother is in a stable state before leaving for the CEmONC (PONEK) facility. 3. Before referrals, primary care should confirm to PONEK Hospital to ensure the availability of appropriate facilities. 4. Health facilities must follow standard operating procedures for patient delivery during COVID-19, including proper infection prevention and control practices. 5. It is necessary to have an accurate, fast and safe referral system (referral manual) that has been agreed between primary care, PONEK hospitals and all related parties to ensure that patients are served immediately and adequately. This includes mapping of PONEK hospitals in each region to ensure the ease and equality of access to referral services in all areas as well as avoiding excess/accumulation of patients in one hospital. 6. The referral system is suggested to be made in the form of policies/regulations from the local health office and be disseminated to all health workers in the region. 7. It is necessary to monitor and evaluate the implementation of the referral system to ensure compliance of all parties and immediate resolution of problems that may occur during referrals. 8. The development of information systems corresponding to the local area can be used to optimize the referral system. |
|  | Preparation of the patient before referral | 1. Prepare transportation equipment and medicines in anticipation of medical emergencies. 2. All workrs who transfer patients must wear appropriate personal protective equipment (level 3) consisting of eye protection (and *face* shields if they are going to perform aerosol-inducing measures), head coverings, *gown all covers*, N95 masks (if available) or layered surgical masks as a second option, sterile rubber surgical gloves, and boots; 3. Patients are asked to wear surgical masks while in transportation (or when entering a health facility) by paying attention to the patient's condition such as oxygenation. 4. Provide equipments and *bag valve mask* (BMV) to reduce aerosolization when the patient's hypoxia worsens during the referrals. |
|  | Vehicle cleaning | 1. Vehicles used to transport patients need cleaning and disinfection internally by officers using PPE 2. Upon arrival at the health facility, the workers who took the patient removed the PPE and disposed of it as directed and washed his hands 3. Ambulance attendants use new PPE before returning to travel on the same ambulance 4. The equipment used while transporting patients is cleaned and/or sterilized according to health facility protocols. |
| Antenatal Service | Risk of Covid in pregnant women | 1. Pregnant women are at risk of developing moderate to severe illness due to COVID-19. Most pregnant women however are expected to experience only mild or moderate symptoms similar to a cold or flu, or sometimes no symptoms at all. 2. Pregnant women have an increased risk of complications from any respiratory disease due to physiological changes that occur during pregnancy. These include a decrease in lung function, an increase in oxygen consumption and a change in immunity. 3. There is no evidence to suggest transmission through breastfeeding, however studies are underway to investigate this further. 4. Babies born from women with COVID-19 have the potential to be infected with the virus after birth (through exposure to droplets), but the risk of transmission can be minimized through general infection control practices. 5. Most infected babies are likely to experience only minor illnesses. 6. Conception products, placenta, amnion, etc. It has not been shown to have exposure or congenital coronavirus infection, and does not pose a risk of coronavirus infection so it needs to be treated as a bloodborne pathogen and handled in accordance with standard waste management practices. 7. Pregnant women may be at risk of experiencing more severe manifestations and sequelae of infection with Coronavirus 2 (SARS-CoV-2) thus allowing comprehensive counseling for pregnant women and their families, in particular regarding the risk of maternal end results at gestational age and the potential risk of intrauterine or peripartum transmission of the virus to the fetus or newborn. 8. Health workers need to continously up-to-date with evidence-based recommendations on maternal health. 9. Based on what is currently known, pregnant people are at higher risk of developing severe illness from COVID-19 compared to people who are not pregnant. There may be an increased risk of pregnancy such as premature birth, among people who are pregnant with COVID-19. 10. The risk of venous thromboembolism has been shown to increase in people with COVID-19 and pregnancy known as hypercoagulation states. |
|  | Determining pregnancy status | 1. Noemally, pregnant women will contact a health facility after learning that they are pregnant to get antenatal care (ANC) or visit a health facility in person to confirm their pregnancy and receive an ANC. The first step in providing maternal and newborn services during the COVID-19 pandemic is therefore to determine the pregnancy status. 2. When a pregnant woman is enrolled in the health service, monitoring is carried out during pregnancy and determination of the approximate date of delivery. |
|  | Monthly monitoring | 1. Once a woman receives a pregnancy confirmation from a health worker, she must receive the first ANC visit standardised from Indonesian Ministry of Health, from a community health worker or visit a health facility to receive an HIV test, tetanus toxoid (TT) immunization, syphilis and Hepatitis B infection tests, blood pressure measurements, urine protein examinations, and assess other chronic conditions or diseases that may affect the pregnancy. 2. At the first ANC visit health workers develop an ANC schedule and examination plan as monthly ANC visits are recommended, but the schedule can be adjusted depending on the context and program/conditions that have changed during the COVID19 pandemic as well as the patient's condition. 3. WHO's recommendation for ANC visits be conducted 8 times while the Indonesian Health Ministry standard was 6 x. Pregnant women who have risk factors may visit more health facilities than the standard. During the pandemic, the meeting can be modified by virtual meetings/consultations. 4. An ANC examination can be carried out by authorized health workers according to their competence such as midwives, general practitioners and obstetrics and gynecologists taking into account the patient's condition; 5. The integrated ANC was carried out once time at the nearest health center. During the pandemic, this can be done by encouraging the mother to arrive early considering the limit of the patient quota per day such as 10-15 people / day (if the quota has been full on that day so it can be scheduled on the next day); 6. Health workers educate all women about the symptoms of COVID-19, the importance of home care, social distancing, wearing masks when meeting with visitors, and maintaining cleanliness at home until delivery unless they experience pregnancy-related complications (including abdominal pain, dizziness, vaginal patches, or bleeding) and/or severe symptoms leading to COVID-19. |
|  | Asking for COVID-19 Symptoms in ANC visit | 1. During pregnancy monitoring, pregnant women need to be evaluated for the possibility of being infected with COVID-19 taking into account suggestive symptoms, contact with family or other people with COVID-19, and the rate of spread of COVID-19 in the community. 2. COVID-19 screening must be conducted to every pregnant woman, especially before or when visiting health facilities. 3. If health workers suspect that a pregnant woman has COVID-19 either because of COVID-19 symptoms, possibly a contact with family or other people with COVID-19, or both, the pregnant woman must be referred for further tests. |
|  | Tracing | 1. Health workers should be vigilant to assess other serious conditions that may cause similar symptoms. 2. When more COVID-19 testing is available, all women should be prioritized to receive a COVID-19 test during routine antenatal care visits at health facilities. |
|  | COVID-19 in pregnant women | 1. Pregnant women with COVID-19 must be closely monitored, and routine antenatal monitoring is still carried out for all mothers because pregnant women have the potential to be infected without symptoms. 2. If necessary, remote monitoring (**monitored daily for 14 days**) and or **homevisit** at the patient's home using appropriate PPE. This can also be conducted through cooperation with the task force (task force) or with the community health workers (cadres); 3. Monitoring of women with COVID-19 can be carried out based on the following classifications: low, medium and high:  - For a mild presentation, women without comorbidities (low risk) should self-isolate at home, - Women with health problems, obstetric problems or inability to take care of themselves (moderate risk) should be evaluated for referral.   The management of confirmed pregnant women is also adjusted to the gestational age and the symptomps severity (Based on Indonesian guidelines).   1. If the woman meets the **self-isolation criteria**, ANC meeting must be rescheduled after the isolation period ends. 2. Women who have symptoms of COVID-19 who experience pregnancy-related complications need to be examined separately from others in the isolation room or at the beginning or end of clinic services when there are no other patients left, to lower the chances of transmission to others. 3. Women with COVID-19 symptoms need to wear masks and maternity care workers should wear PPE according to WHO recommendations 4. **All confirmed women, both referred and self-isolating, need to be recorded and monitored regularly.** |
|  | Clinical management of pregnant women affected by Covid | 1. According to The Royal College of Obstetricians and Gynecologists (RCOG), pregnant women with moderate symptoms should self-isolate, unless they attend a maternity unit in which patients in the 2nd or 3rd trimester meet the riteria ( ≥ 1 of: (1) Clinical / radiological evidence of pneumonia, (2) Acute Respiratory Distress Syndrome (ARDS), (3) Fever ≥37.8 and at least one of acute persistent cough, hoarseness, nasal discharge/nasal congestion, shortness of breath, sore throat, wheezing or sneezing), should be checked for COVID-19 and treated as infected until the results appear. 2. If pregnant women come with severe symptoms (high risk), they should be taken immediately to **the emergency department / hospital (PONEK) which is also registered to handle** **COVID-19** cases**.** 3. The woman should be immediately taken to an isolation ward or a designated clinic room, which should be appropriate for most of her treatment while in the hospital. Where the women should not remove her face mask until she is isolated and alone in the appropriate room or group. 4. Women with suspected COVID-19 infection should be placed in a separate room from confirmed cases until a confirmed test result is obtained 5. For women who have not previously been registered with COVID-19 who come with serious obstetric complications (e.g. uterine rupture, etc.) who require immediate surgical intervention, the delivery attendant must follow the general provisions for providing care to women suspected/confirmed COVID-19 and wear the appropriate PPE for the surgical procedure 6. **There should be regular drills/practice to prepare** and build confidence of the workers, and identify deficiencies to prepare for emergency transfer to the operating room. 7. Women without COVID-19 symptoms:   If a woman is not experiencing symptoms of COVID-19, then she should be referred to a PONEK referral health facility for regular ANC visits as per the routine care.   1. Women with COVID-19 symptoms:  - If a community health worker during the initial examination finds that a woman has symptoms leading to COVID-19 or recent close contact with someone with COVID-19, the health worker should refer the woman to a central COVID-19 testing and treatment immediately. The woman must receive information about self-isolation while waiting for the test result, and if the test is positive, she must follow quarantine guidelines. All women who test positive for COVID-19 should follow up contact tracing in the community. - If a woman shows symptoms of COVID-19 during an examination in a health facility, she should be referred to a treatment center for a COVID-19 test as soon as possible, after which to carry out an examination for other conditions that may appear with similar symptoms. - If at any point during the initial ANC or pregnancy status check, a woman shows severe symptoms corresponding to COVID-19 (in particular, difficulty breathing; persistent pain or pressure in the chest; new confusion; inability to wake up or stay awake; bluish lips or face; fever> 38.6 ° C; or severe cough, nausea, vomiting, or diarrhea), she should be immediately referred to a COVID-19 treatment center for treatment. - **Clinical management should consider gestational age including considerations for termination or maintaining pregnancy according to the women’s condition.** |
|  | Prone position in pregnant women to relieve symptoms. However, there are limitations when this is conducted in primary care | 1. Consider a periodical prone position for pregnant women on a mechanical ventilator due to COVID-19 2. Consider the prone position for pregnant and postpartum women with COVID-19 symptoms who get all forms of oxygen therapy and have not been intubated. 3. When positioning a pregnant woman in a prone position, care should be taken to support pregnancy in the uterus to reduce aortic-caval compression. 4. The birth of the baby should be considered when it can increase the resuscitation of the mother or benefit the fetus. 5. Avoid abdominal compression and ensure that the woman's hips and chest are supported. In the absence of special equipment, prone can be carried out using pillows and blankets. |
|  | Vaccination in pregnant women | As per the routine practice to Indonesian antenatal care guideline. Women with COVID-19 should delay the vaccination until cleared from COVID or according to the advice of obstetrician. |
|  | Telemedicine/remote monitoring | 1. With the availability of digital technology services, some ANC appointments can be made remotely using telehealth, such as by phone or video chat (long-distance contact), to ensure that there is no disruption to the mother's pregnancy. 2. Doctors/Midwives need to use clinical judgment when deciding which mother with COVID-19 is suitable for an alternative treatment schedule that includes remote prenatal visits. 3. Health information provided by the midwife or other maternity service provider at the beginning of the antenatal clinic should include information on social distancing in the clinic (i.e.: sitting two arms apart from each other) and important messages about the virus (such as symptoms, home isolation procedures, emergency signs, etc.). 4. Information sessions can be used as an opportunity to minimize women's fears about the impact of COVID-19 on pregnant women and newborns and advocate ongoing communication with pregnant women. 5. Follow-up by phone and or video during postnatal may be considered to replace postnatal care visits at health facilities, if no cprocedures or physical examinations are required. 6. **Community health workers should identify and record women who gave birth at home at the time of their visit.** 7. The availability and incorporation of telemedicine has increased dramatically during the COVID-19 pandemic, and has proven to be an important complement to patients requiring visits, along with multi-disciplinary combined visits for indication of routine and higher-risk pregnancies, and antenatal limiting 8. The use of other audiovisual meeting platforms during the visit period and in the examination room is equipped with a smartphone stand when necessary to allow clients to share and actively participate, or indoor meetings that allow social distancing. 9. Barriers to accessing telemedicine should be assessed, including lack of access to technology or the Internet, security of patient data/information, lack of privacy due to housing constraints, concerns about partner violence, or the presence of hearing loss. |
|  | Involving the community / Cadres | Develop a sustainable ANC service model for the context of society:   1. Establish mechanisms to ensure that there is coordination of care across an ANC's touchpoints, including community-to-facility relationships and oversight that support community-based services, activities and additional health workers. 2. The empowerment of health cadres/additional officers in the community should begin with socialization/training to ensure that all activities carried out meet health protocols. 3. Support the reorganization of ANC services and/or client flows, as needed, to reduce waiting times and contact with other patients, improve service delivery efficiency, and satisfaction between clients and providers. 4. Limit the number of women visiting in the clinic in each day. |
|  | Other examinations, such as ultrasound and XRay | 1. Radiological examination should be carried out in accordance with the guidelines. 2. Ultrasound examination should not be postponed when it is urgently needed during maternal health monitoring. 3. Women who have confirmed COVID-19 are referred for antenatal ultrasound services to monitor the fetal growth after symptom determination. 4. Women are also advised to take ultrasound at the 1st and 3rd trimesters. 5. Radiological investigations should be carried out in accordance with the guidelines. If there are concerns about the safety of the fetus should not delay the examination. 6. Chest imaging (X-ray or CT) with abdominal protection can be conducted because it is important to examine patients with unhealthy COVID-19 and should not be delayed as it concerns the fetus. 7. Additional examinations such as blood clotting profiles, electrocardiograms, CT pulmonary angiography, and echocardiograms can be performed to determine differential diagnosis. 8. Pulmonary embolism should be considered if there is chest pain, worsening hypoxia (especially with a sudden increase in oxygen demand), or when shortness of breath persists or worsens after the expected recovery from COVID-19. |
| Labor | Screening before of labor | 1. All pregnant women should be guided to prepare and plan their delivery (P4K program) according to their conditions: place, helper, delivery financing as well as blood donors, transportation and necessary equipment. 2. All pregnant women are informed about the signs of childbirth and should visit immediately to a health facility in case of such signs. Mothers with high risk of pregnancy complication must be educated to go directly to the PONEK facility for the delivery process in accordance with P4K. 3. Health officers should periodically identify the Estimated Day of Birth (HPL) of pregnant women in their area to prepare adequate facilities. 4. Access to delivery room should be limited to ensure that all visitors and patients have been screened. |
|  | Fetal Monitoring ahead of delivery in pregnant women with COVID/ suspected COVID | 1. Electronic Fetal Monitoring (EFM) **is recommended during childbirth for all suspected or confirmed cases of COVID-19.** 2. If a continuous Electronic Fetal Monitoring (EFM) facility is not available, monitoring uses a Doppler or by means of manual auscultation of the fetal heart rate or must be performed during labor, as indicated for high-risk childbirth. 3. For women recovering from COVID-19 infection, ongoing monitoring of fetal growth, especially during the third trimester is needed to determine the impact of COVID infection on the fetus or placenta. |
|  | Decisions of birth | 1. The decision to baby delivery must take into account the gestational age, motherhood, and fetal status and it is necessary to stabilize the women before delivery. 2. **The mode of delivery in pregnant women infected with COVID-19 should be based on an assessment of their obstetrics and physiological stability (cardiorespiratory and oxygenation status). COVID-19 infection itself is not an indication of induction of labor or operative labor.** 3. Patients with impaired cardiopulmonary function requiring intubation, child-birth at the age of 32 weeks should be considered and the decision should be balanced with the benefits of continuation of pregnancy after consultation with a senior obstetrician and neonatologist. 4. Delayed cord clamping is part of standard care, regardless of the presence of COVID-19, there is currently no evidence that delayed cord clamping affects the risk of vertical transmission of COVID-19. 5. The current evidence does not support a specific way of birth, but water birth should be avoided if the woman has been suspected or confirmed with COVID-19 6. A positive outcome of COVID-19 in asymptomatic women where there is no evidence of harmful fetal disorders is not an indication to accelerate the childbirth. 7. The way of childbirth should be discussed with the expectant mother, taking into account the preferences and obstetric instructions for carrying out the action. 8. The mode of delivery should not be affected by a positive outcome of COVID-19, unless the woman's respiratory condition demands immediate action for delivery. The decision on the way of birth should not be affected by the presence of COVID-19, unless there is an indication of a maternal or fetal emergency as in the usual practice. 9. When a cesarean section or other surgical procedure is required, follow the infection control guidelines by using the appropriate PPE 10. Care during childbirth should not differ from usual, however given the relationship of COVID-19 with acute respiratory distress syndrome, women with moderate-severe SYMPTOMS of COVID-19 should be monitored using an hourly fluid input-output graph, plus targeted efforts to achieve fluid balance during labor, to avoid the risk of excess fluid. 11. There is currently no evidence to suggest that cesarean section in women with COVID-19 can reduce the risk of vertical transmission to newborns so the way of birth should be continued as usual care. |
|  | Triage during the labor | 1. All patients who are about to deliver the baby are screened for COVID-19 from the age of >37. Such screening needs to be reconfirmed (if possible) when the women comes back to the health service at the time of delivery. 2. The type of COVID-19 screening test is adjusted to the policy and the availability of tools and materials. A positive maternal test result on atibody or antigen screening needs to be confirmed with a PCR test (if available). 3. COVID-19 screening can be carried out at Puskesmas and in midwife practce. 4. If the women turns out to be positive for COVID-19, measures must be taken in accordance with the Ministry of Health Guidelines for detecting suspected/confirmed cases of COVID-19 in non-COVID health facilities 5. Reception and triage should be in the same room that will be used for entry in the delivery room, which is ideally a low-pressure room. 6. Keeping the room free of unnecessary items (decorations, extra chairs, etc.) that can later become infected as well. 7. Pregnant patients with suspected or confirmed COVID-19 must notify the workers prior to arrival so that the health facility can direct the patient to PONEK or prepare for proper infection control such as: identifying the most appropriate space for delivery, ensuring infection prevention and control and PPE are placed correctly, and notifying all health workers who will be involved in patient care on infection control before the arrival of the patient.   **Primary care is directly referred to the PONEK hospital if the patient is COVID-19 positive.** |
|  | Acceleration of labor | 1. Consideration can be given to shortening the second stage with pervaginam delivery in patients who meet prerequisites including a fully widened cervix, fetal head involvement, low fetal standing, adequate clinical pelvimetry, and patient consent. 2. If possible, women affected by Covid, straining during birth should be minimized. Deep breath efforts and those that mothers emit can increase visitors' and staff's exposure to the patient's respiratory secretions. |
|  | The need for isolation room | 1. Low-pressure isolation rooms are more suitable for patients who require aerosolization procedures (breathing apparatus, suction, nebulation). If unavailable, low pressure can also be created by an exhaust fan that pushes air out of the room. 2. The isolation room must have adequate ventilation. If the room is air-conditioned, ensure air change and exhaust air filtration /12 hours. This area should not be part of the central air conditioner. 3. In a health facility, the waiting room must be a well-ventilated room (or open space) where other outpatient patients/families can keep their distance and wear masks. 4. The majority of women who give birth will not experience respiratory symptoms, and the delivery room can provide services as before. However, attention to infection prevention practices should:  - Have sufficient supplies for all PPE supplies (masks, gloves, goggles, protective gowns, hand sanitizer, soap and water, cleaning supplies) in the workspace - All surfaces should be thoroughly cleaned with a spray and a clean cloth upon contact with the patient or attendant - Attendants should practice regular hand hygiene - wash their hands before and after examining each patient.  1. It is necessary to carry out permanent restrictions on companion persons (1 person desired by the woman) while in the isolation room,. The companion has to be screened to ensure that he has no symptoms or risk of exposure to COVID-19 and has been educated about any impact of exposure that can occur. 2. The use of special *delivery chambers* has not been recommended. 3. The implementation of emergency delivery of COVID-19 suspect/confirmed patients in primary care should be prepared by paying attention to the prevention and control of high levels of infection. |
|  | Discharge Procedure | 1. Women who are positive for COVID-19 or with symptoms corresponding to COVID-19 infection should remain in a health facility after giving birth until the symptoms disappear. 2. In the event of an outbreak or exposure to COVID-19 in a health facility while the woman is staying, the length of stay in the health facility (e.g. 24 hours) may be shortened to reduce the potential exposure to COVID-19 3. Discharge after 6 hours in women and newborns born pervaginam without complications can be made provided that women have support at home, with a continoues remote follow up from maternity service personnel. |
|  | Possibility of vertical transmission during childbirth | 1. Vertical maternal and neonatal transmission is suspected to occur in some confirmed COVID-19 cases with positive RT-PCR or an increase in anti-COVID-19 IgM and Ig-G levels at birth where pneumonia is the most common infection, in addition to fever and gastrointestinal disorders. 2. However, COVID-19 is considered mild in most neonates with only a few neonates requiring short-term respiratory assistance and all allowed to go home from the hospital. |
|  | Is it necessary to separate the room | 1. Mothers who test positive for COVID-19 and healthy babies do not need to be separated. 2. Given the limited evidence, women with COVID-19 and their healthy babies, who do not require neonatal care, should stay together immediately after delivery minimizing pressure and ensuring mother-child bonding and adequate breastfeeding or may request helpfrom neonatologists and families for neonatal care. 3. The risk of separating mother and baby to reduce the transmission of infection and the potential for mild disease in the baby, may far outweigh the benefits of keeping mother and baby for direct skin-to-skin contact and early initiation of breastfeeding for thermal regulation, prevention of hypoglycemia and reducing sepsis and death in infants. 4. All mothers and babies regardless of COVID-19 status, need support to stay together to practice joint care, breastfeeding practices, practicing skin contact or kangaroo mother care. 5. Every newborn has the right to access the mother or his parents so that the mother who should not be separated from her baby without her consent. 6. Neonates who are symptomatic/sick and born to a suspected or confirmed mother with COVID-19 should be treated in a separate isolation facility from the rest of the baby. 7. Patients who can be discharged from the hospital but have not met the criteria to stop isolation and want to reduce the risk of transmission to newborns can continue temporary separation at their place of residence (if possible) until allowed to stop self-isolation based on symptom strategies or examination results 8. If considering a temporary separation, the risks and benefits should be discussed by the mother and a team of health workers. Where the decision on a temporary separation should be made according to the wishes of the mother. For more information, see the guidance in Home Isolation Stoppages for People with COVID-19. |
|  | When the baby has to separate from his mother | 1. If there are not enough rooms available, a closed incubator or luminous warmer can be placed in a general isolation ward for neonates with a neonatal bed distance of at least 1 meter from each other 2. Suspected and confirmed cases of COVID-19 should ideally be managed in separate isolation. If it is not possible to have separate facilities and suspected and confirmed infected neonates are in one isolation facility, they should be deserted by providing sufficient space for both groups 3. The isolation ward should have a separate double entrance with a dressing room and a treatment room. The premises should be away from the NICU/SNCU/maternity room/postnatal ward. |
|  | Skin-to-skin contact of women and baby | 1. Skin-to-skin contact from birth and during the postnatal period regardless of the presence of COVID19. However, parents with COVID-19 should use infection prevention and control measures (mask and hand hygiene) 2. Skin-to-skin contact should be recommended and resumed as usual in other postpartum and neonatal environments, such as neonatal intensive care units and postnatal wards, provided that infection prevention and control measures are maintained. |
|  | Anxiety in pregnancy | 1. The COVID-19 pandemic increases the risk of antenatal anxiety and depression as well as domestic violence due to the economic and social impacts of the COVID-19 pandemic so that health workers need a guidance / referral mechanism to support them. 2. Postnatal anxiety and depression are common in mothers as well as many new fathers who may be exacerbated by social isolation and the financial impact on families and communities due to the COVID-19 pandemic. 3. Health workers are encouraged to cope with stress in patients during the COVID-19 pandemic as mothers caring for infants and young children may experience increased stress, feelings of isolation, or loneliness due to social distancing measures during COVID-19 outbreaks or temporary separations and symptoms of postpartum depression may worsen due to COVID-19 social distancing. 4. Health workers should inquire about mental health during each consultation and connect women with relevant resources, if needed. Where ordinary referrals should not be delayed. 5. Integrated ANC services allow healthcare workers to work in teams to address physical and mental issues. In primary care, pregnant women at risk of anxiety and depression can be directed to clinical psychology (if any) or consulted to a general practitioner or mental program holder at a health center. 6. Patients who are confirmed with COVID-19 may have higher anxiety level so the management approach needs to be more intensive |
| Use of medication | Use of NSAIDs | 1. There is no evidence that NSAIDs worsen disease conditions in COVID-19 patients. 2. Several studies have shown the benefits of using NSAIDs where a 2006 study showed that indomethacin actually has antiviral activity against SARS-CoV. 3. The choice of postpartum analgesics, we recommend continuing the use as indicated clinically |
|  | Use of ACE in postpartum | There is currently no harmful evidence of ACE inhibitors therapy for postpartum women with COVID-19 suffering from hypertension unless there are contraindications. ACE inhibitors are contraindicated in the antenatal period because they can harm both the fetus and neonatals. |
|  | Aspirin Use | As per routine indication for prevention of preeclampsia. |
|  | The use of heparin for the prevention of severe COVID (in Hospital) | 1. All pregnant women and postpartum mothers must undergo an examination of VTE (venous thrombolis) risk factors upon hospital admission and after delivery., whether diagnosed with COVID-19, whether there is a change in the severity of COVID-19. 2. The use of prophylactic pharmacology in women should be accompanied by other measures to prevent VTE, such as anti-embolism stockings and a series of compression devices. 3. Using prophylactic dose anticoagulants, especially low molecular weight heparin (for example, enoxaparin 40 mg once a day or dalteparin 5000 IU once a day) for pregnant women or postpartum mothers who are hospitalized (with any indications) and who have COVID-19, unless there are contraindications, such as the risk of major bleeding or immediate delivery. Where anticoagulant prophylaxis should be continued at least 14 days after discharge from the hospital or until the thing of abnormalities related to COVID-19 (immobility, dehydration and / or shortness of breath) has been resolved. 4. Consider increasing the administration of prophylactic anticoagulant doses, preferably *Low molecular weight heparin* (eg, enoxaparin 40 mg twice a day or dalteparin 5000 IU twice a day) for pregnant women with severe or critical COVID-19, or where there are additional risk factors for VTE, unless there are contraindications, such as the risk of major bleeding or platelet counts <30 × 109 / L. Where prophylactic anticoagulants should be continued for at least four the week after discharge from the hospital or until COVID-19 related abnormalities (including immobility, dehydration and/or shortness of breath) have been resolved. 5. The dosage *of low molecular weight heparin* depends on the weight before pregnancy and current kidney function. For women with an early body weight of pregnancy beyond 50-90 kg, consideration of the dosage of *Low molecular weight heparin* needs to be adjusted. 6. In some situations, a follow-up *low molecular weight heparin* is required during post-pregnancy and postpartum. 7. Requires the involvement of obstetricians, obstetricians, hematologists or other doctors with expertise in VTE in pregnant women. 8. Consider the administration of prophylactic dose anticoagulants, it is better that LMWH (for example, enoxaparin 40 mg once a day or dalteparin 5000 IU once a day ) for pregnant or postpartum women who self-isolate at home with mild COVID-19 and there are additional risk factors for VTE, unless there are contraindications, such as the risk of major bleeding or childbirth 9. Considering for prophylactic administration of anticoagulants at least 14 days dose, preferably LMWH (i.e., Enoxaparin 40 mg once a day or dalteparin 5000 IU once a day) unless there are contraindications, such as the risk for major bleeding. 10. Consider an increase in duration for six weeks if COVID-19 gets worse or critical and/or additional risk factors for VTE. 11. Pregnant women suspected or positive for COVID-19, especially with hypoxia, prophylactic administration of VTE (venous thrombolis) can be stopped after discharge from the hospital unless medically indicated otherwise |
|  | Use of prostaglandins | 5-Methyl prostaglandin F2α is a commonly used uterotonic for postpartum hemorrhage and should be used with caution in patients with suspected or confirmed COVID19 infection due to its ability to increase vascular resistance of the lungs so the drug is contraindicated in patients with active respiratory diseases, such as patients with severe COVID19 infection. |
| Breastfeed | The importance of breastfeeding | 1. The benefits of breastfeeding today outweigh the risk of transmitting the infection from mother to baby. 2. Maternity care providers need to support the mother to breastfeed and if the mother is unwell can provide support to the mother to squeeze the milk and give it to the baby. 3. It is known that there are many known benefits of breastfeeding, mothers should be supported to start or continue breastfeeding so that if the baby is breastfed or formula milk, infection prevention and control measures must be taken because there is currently no evidence to suggest that breastfeeding increases the risk of vertical transmission to newborns. |
| What if the woman/child gets Covid or is a close contact of a COVID? | Breastfeeding women affected by COVID | 1. If a nursing person experiences symptoms of COVID-19 or receives a positive virus test result, they should continue breastfeeding precautions. 2. There is no evidence of beneficial effects or risk hazards of COVID-19 after direct breastfeeding or dairy feeding. 3. Stable neonates who are exposed to COVID-19 and are in the room with their mother can be discharged along with the mother's discharge and continue self-isolation. 4. Stable neonates who cannot be hospitalized due to their mother's illness and will be treated by a trained nurse or family member, can be discharged from the facility at the age of 24-48 hours. 5. Returning home early may be followed by a follow-up by phone or a home visit by a designated health care worker as there is no evidence of any beneficial or harmful impacts with early discharge after exposure to COVID-19. 6. Mothers and family members should be counseled on the red flags and advised to report back to the health facility if the neonate shows the development of such red flags, |
|  | Both woman and baby have COVID-19 | 1. Breastfed mothers and breastfed children with suspected or confirmed Covid1-9 should follow self-isolation protocols and avoid breastfeeding in the breast by squeezing milk and feeding milk from bottles during the self-isolation period. 2. If the symptoms are more severe, then follow the applicable patient handling procedures. |
|  | Neonates/children including close contact of mothers affected by covid | 1. A child breastfed by someone suspected or confirmed with COVID-19 should be considered a close contact of someone with COVID-19, and should be quarantined during a period of self-isolation as recommended in nursing parents and during their own quarantine period thereafter. 2. A child who is breastfed with a suspect or confirmed COVID-19 must follow the advice on self-isolation. 3. People breastfeeding a child suspected or confirmed with COVID-19 should be considered a close contact of someone with COVID-19, and should be quarantined during the recommended self-isolation period for the breastfed child and during their own quarantine period thereafter. 4. Neonates who are directly exposed to close contact with those infected with COVID-19 (including family members, caregivers, medical personnel, and visitors) should be managed as patients in the investigation regardless of whether they are symptomatic or not. |
|  | Both woman and baby are close contacts | If the nursing mother and the breastfed child have been in close contact with someone who is isolating and quarantining due to COVID-19, then:   - Breastfeeding mothers and breastfed children should be quarantined after their last contact with a person who has COVID-19. - If one or both experience symptoms, or receive a positive virus test result, the person must follow the rules for home isolation. - If only one of them experiences symptoms or receives a positive virus test result, one of the others that is not infected should be quarantined according to the recommended period of isolation at home and during the self-quarantine period thereafter. - If a nursing person experiences symptoms of COVID-19 or receives a positive virus test result, they should continue breastfeeding precautions. |
|  | The child is in close contact with COVID-19 patient but the breastfeeding woman is not in close contact | If the breastfed child has been in close contact with a person who has COVID-19 other than the nursing mother (for example, another nanny, a nanny), but the nursing mother has not been in close contact with anyone who has COVID-19, then:   - Breastfed children must be quarantined after their last contact with a COVID-19 case. - People who are breastfeeding should be monitored for signs or symptoms of COVID-19 but do not require quarantine unless the nursing child develops symptoms of COVID-19 or receives a positive virus test result. - Avoid breastfeeding in the breast by squeezing milk or giving milk from the bottle. - Use a mask during close contact (that is, less than 6 feet) with the child and clean hands frequently (that is, before and after touching the child). |
|  | Monitoring for postpartum women | Postpartum women who are confirmed with COVID-19 can be monitored using telemedicine during the self-isolation period (if there are no problems/difficulties) and afterwards can visit health facilities or can be visited by health workers (*homevisit*) according to the standards of postpartum visit/*Kunjungan Nifas (KF)*. |
|  |  | The examination of puerperal woman is carried out according to the standards of KF 1, 2, 3, and 4 examinations (according to the guidance frorm Indonesian Ministry of Health) by considering the patient's condition and status and adhere to the infection control and prevention procedures. |
|  | Family support | 1. Patients with COVID-19 can be discharged from clinically instructional health facilities. 2. Family/caregiver support is needed for pregnant women with COVID-19. |
|  | Education to the public | 1. If the women come to a health facility as an outpatient for postnatal care, the waiting room should be a well-ventilated room (or open space) where other outpatient patients/families keep their distance and wear masks. 2. If the women’s COVID-19 progression gets worse during the postpartum period, the mother should be referred to a designated COVID-19 care center. 3. Training on infection control and prevention measures is provided to all cadres and health workers based on proficiency before starting service activities. 4. Community-based contact tracing should be done for all mothers who test positive for COVID-19. |
| Postpartum and well-baby visits | General principles | 1. Postpartum mothers are still monitored using a postpartum checklist or applicable monitoring. 2. Healthcare providers are encouraged to prioritize newborn care and vaccinations 3. During the visit, health workers should evaluate feeding and weight gain (especially given the potential for breastfeeding disorders due to COVID-19 disease), assess dehydration and jaundice, assess stressors and caregivers' caregivers, and provide appropriate support 4. Breastfeeding mothers should be counseled to inform their child's health services that their child, or their child's caregiver, has been in close contact with a person suspected or confirmed to have COVID-19 prior to a treatment visit or if the child has developed symptoms of COVID-19. 5. Healthcare providers should consider how to minimize exposure to COVID-19 for patients, caregivers, and health workers in the context of local COVID-19 epidemiology and practice environments. 6. During the COVID-19 pandemic, it is imperative to ensure that people who are breastfeeding or who want to breastfeed continue to have access to professional lactation support (e.g., lactation consultants, pediatric or obstetric health care workers) |
|  | Visiting health centers | 1. In-person visits are only scheduled for those with negative COVID-19 symptoms at the time of making an appointment visit. 2. **The minimum standard of postpartum (KF) is carried out according to standards, namely KF 1** (6 hours to 2 days postpartum), KF 2 (3-7 days postpartum); KF 3 (8-28 days postpartum day), KF 4 (29-42 days postpartum) based on the Indonesian maternal guideline. 3. At the time the patient is pregnant, in society the regulations on restrictions on activities outside the home and social distancing are sometimes arbitrary so it is necessary to limit visits to health facilities. 4. It did not find any rejection of symptom screening by patients at the time of scheduling a visit, or at the time they came for an appointment, before entering the formal office area. |
|  | Vaccination | 1. Follow routine immunization policies in healthy neonates born to mothers who are suspected / proven to be confirmed with COVID-19 2. In neonates who are suspected / confirmed to be infected, vaccination must be done before discharge from the hospital according to the applicable policy |
|  | Breastfeeding consultation    Note: Breastfeeding counselors are still rare in Indonesia, potentially be conducted during home visit postpartum | 1. Breastfeeding counselors need to be given updated training related to their work and also related to maternal assistance during the pandemic. 2. Breastfeeding consultations usually require close contact between a lactation specialist and a nursing child-caregiver; therefore, the proper use of personal protective equipment (PPE) is essential. 3. During the COVID-19 pandemic, if necessary, breastfeeding consultations use alternative approaches, such as telemedicine, to provide lactation support services whenever possible, especially when providing support for breastfeeding mothers suspected or confirmed COVID-19 4. Breastfeeding counselors working in health care settings should follow the recommended infection prevention and control measures for health care establishments (e.g., hospitals, clinics, doctor's offices) 5. Breastfeeding counsellors provide home visits, nursing mothers receiving visits and other home members should protect themselves from COVID-19 as recommended (before entering the home it is necessary to take precautions and control of infections) 6. Breastfeeding counselors should stay home if they are sick with COVID-19, think they may have COVID-19, or have been in close contact with someone who has COVID-19 7. Refer all clients to another counselor until the health worker meets the criteria for returning to work after sars-CoV-2 infection is met 8. Screening clients by phone to find out the latest symptoms of COVID-19 and exposure for people diagnosed with COVID-19 before making a home visit. 9. If anyone in the house has been suspected or confirmed to be COVID-19, it is recommended that lactation services be provided via telemedicine during the self-isolation period in accordance with the recommendations coupled with an additional quarantine period recommended for other family members. 10. If a client or other family member has COVID-19 and at-home services are deemed necessary and critical, use all recommended personal protective equipment as described in the Infection Control Guide for Healthcare Professionals on Coronavirus (COVID-19) in treating patients suspected or confirmed with COVID-19. 11. If a client or family member is not known whether COVID-19 is suspected or confirmed, wear a mask while inside the client's home and dispose of a disposable surgical mask on each client. 12. In communities with moderate to high transmission, consider wearing eye protection in addition to surgical masks to ensure that the eyes, nose, and mouth are all protected from exposure to respiratory secretions when providing breastfeeding assistance. 13. For all home visits regardless of the client's or family's COVID-19 status, it requires the client as well as other family members aged 2 years and older to wear masks. 14. When not providing direct observation, keep at least 6 feet away from clients and other members at home. 15. Wear a mask at all times and even more importantly if the distance is less than 6 feet. 16. Use disposable gloves when touching a client or child. 17. Wash hands with soap and water for at least 20 seconds when entering and leaving the house. |
| Visitor Restrictions during the ANC and postpartum visit | Visitor restrictions | 1. Patients, accompanying persons and attendants in the examination room should be limited. 2. The patient's introduction is limited to the closest person and preferably the same person during treatment, for example when the patient gives birth. 3. It can be considered to use of video/teleconference to connect patients and their families. 4. Enough one companion during childbirth. 5. If COVID-19 cases rise, no family visits may be allowed to accompany the women. 6. The length of patient visits also needs to be limited. 7. For uncomplicated childbirth patients, the examination should be spaced out to limit the attendants who are in direct contact with the patient. 8. The husband/one family person is allowed to live with the woman, during childbirth and birth because the constant support of the couple is known to increase spontaneous pervaginam births, shorten childbirth and reduce cesarean births and other medical interventions. 9. If a husband/immediate family shows symptoms, they should remain self-isolating and not attend labor and be notified for the preparation of alternative birth support possible if needed |
|  | Visitor rules | - Visitors should be informed about the use of masks (including cloth face coverings), social distancing for everyone entering the health facility and about the proper use of personal protective equipment in accordance with the current policy for visitors to the health facility. - Visitors should be instructed to only visit the patient's room and should not go to other spaces in the health facility, including the newborn nursery. |
| Self-Protection🡺 proposed to be the Use and Management of PPI Facilities | General Protection  Personal Protective Equipment  Cleaning procedures  Equipment Management  Physical Facility Management | 1. Consider the availability of health workers and the transmission rate of Covid in the workplace. 2. Actions for patient:    1. All staff and patients need to have access to handwashing facilities and are encouraged to do so when they enter health facilities. Ensure a clean supply of water (even from a bucket if running water is not available), at each location or room where attendants work and in the patient's waiting room.    2. Ensure the availability of soap in every wash in the health facility and a clean cloth or disposable towel for drying hands.    3. Midwives who provide direct care to patients need to wash their hands frequently with soap and water: Hands should be thoroughly washed with soap and water for at least 20 seconds. Wash hands before each patient and before the physical examination. Wash hands again immediately after the examination and after the patient is gone. Wash hands after cleaning surfaces. Wash hands after coughing or sneezing. Hand sanitizers can also be used, especially as a backup for unreliable water sources.    4. Avoid touching your eyes, nose and mouth.    5. Advise everyone (patients and attendants) to cough into their tissues or elbows and to wash their hands after coughing and sneezing.    6. Midwives should maintain social distancing of as many as 2 arms as long as possible during clinical meetings. Physical examination and patient contact need to be continued as usual for women without suspicion / confirmed COVID-19 so that hand washing is done before and after. 3. Healthcare providers must wear appropriate personal protective equipment when treating suspected or confirmed patients with COVID-19. 4. Universal Precaution standards to prevent contact with body fluids, in addition to use personal protective equipment (PPE) such as covering masks such as N95 and face shields with faceshields or at least glasses to prevent contracting infection through respiratory droplets. 5. There must be facilities for health care workers to safely remove and dispose of PPE at the exit of the room where the patient is being treated. 6. All health workers treating patients confirmed positive for COVID-19 must wear appropriate personal protective equipment (PPE). 7. Vigilance for droplets and contact (including face shields or goggles, surgical masks, protective gowns, and gloves) should be used for all clinical interactions in accordance with the guidelines of the Centers for Disease Control and Prevention (CDC) and the World Health Organization (WHO) 8. N95 masks should be used whenever an aerosolization procedure is being performed or is likely to be performed on patients with suspected or confirmed COVID19 such as cesarean, postpartum hemorrhage requiring transfer to the operating room, or intubation. 9. Maternity care providers involved in direct care of patients should have access to PPE. 10. For maternity care services that provide care to women with suspected or confirmed cases of coronavirus in health facilities, the following PPE needs to be worn: long-sleeved gowns, surgical masks (for all patient interactions) or N95/P2 masks (if maternity care services are directly involved in aerosol procedures such as suctioning airway secretions, administration of nebulizing drugs or CPR), eye protection and non-sterile gloves. 11. For maternity care services that provide care to women without coronavirus symptoms at health facilities WHO recommends that PPE needs to be used in accordance with prevention and risk monitoring standards. 12. Using PPE for all patient contacts will depend on the availability of PPE in health facilities and the determination of the risk of exposure by maternity care services. 13. Plastic gloves and aprons need to be used during the transfer of the patient which may involve exposure to blood, body fluids, secretions, excretions, touching the oral mucosa, or treatment assistance (including: taking blood or vaginal swabs, performing stretching and sweeping and the first stage of labor). 14. All patients and visitors should be encouraged to always wear surgical masks, especially those suspected or confirmed to be infected. 15. Using personal protective equipment before disinfecting 16. If the equipment or surface appears dirty, first wipe it with soap and a suitable solution of water or wet cloth before using the disinfectant 17. 0.5% sodium hypochlorite (equivalent to 5000 ppm) can be used to disinfect large surfaces such as floors and walls at least once per shift and for cleaning after the patient has been removed from the area. 18. 70% ethyl alcohol can be used to disinfect small areas in each use, such as special reusable equipment. 19. Hydrogen peroxide (dilute 100 ml of H2O2 10% v / v solution with 900 ml of distilled water) can be used to clean the surfaces of incubators, open treatment systems, infusion pumps, scales, spare equipment. 20. Follow the guidelines for handling routine biomedical waste disposal, sorting, transport and final disposal as specified. 21. Consider infection prevention and control for health facilities that provide obstetric care for pregnant patients with suspected or confirmed coronavirus disease (COVID-19) in inpatient obstetric health services including obstetric triage, childbirth and childbirth, recovery and inpatient postpartum arrangements. 22. Because maternity care units vary in physical order, each facility must consider the appropriate space and staff needs to prevent transmission of the virus that causes COVID-19. 23. These considerations include the appropriate isolation of pregnant patients who are suspected or confirmed with COVID-19; basic training and refreshment for all health care personnel in those units to include proper adherence to infection control practices and the use and handling of personal protective equipment (PPE); and adequate and appropriate supplies of PPE are placed at all points of care. 24. Health care providers should immediately notify infection control officers at health facilities about the anticipated arrival of suspected pregnant patients. 25. A COVID zone is a separate area with an isolated room intended for the care of women who are suspected or confirmed with COVID-19 so must have its own supply of materials and equipment that should not be moved or used outside the zone unless necessary |
|  | Practice site cleaning | - During each period of contact with the patient, maternity care providers are recommended to use routine infection prevention and control practices, such as hand washing. - It is advisable to clean the outside (the exposed) with a cleaning product (ex: 5% sodium hypochlorite (bleach)) and wipe it with a paper towel or clean cloth and it is necessary to follow it by washing hands from one patient to another. - In addition to infection control practices, maternity service providers need to maintain as much as 2 arms as far apart as possible from others to further reduce the risk of infection. - Physical examination should be maintained by washing hands before and after contact with the patient. - Surfaces used by patients and staff need to be sprayed with cleaning products (ex: 5% sodium hypochlorite (bleach)) and wiped with paper towels or clean cloths on each patient, followed by hand washing. |
| Multidisciplinary team | The importance of a multidisciplinary team | - The main action planning meeting by a multidisciplinary team ideally involves the following personnel: consultant doctors (infectious disease specialists or clinical microbiology), obstetric consultants, senior midwives, neonatologists, and anesthesiologists responsible for obstetric care (in hospitals).  **In**  primary **care, the multidisciplinary** **team can involve general practitioners, laboratory officers, nurses, dentists, clinical psychologists, as well as obstetrics & gynecologists, pediatricians (in** **the context of referrals or consultations) and non-health such as health cadres .** - The discussion should be communicated to the expectant mother and the following should be considered: top priorities for maternal and fetal /neonatal medical care, birth preferences, the most appropriate location of care (e.g. intensive care unit, isolation) - The disciplinary team should perform an examination of whether it is an emergency delivery or induction of labor, either to assist the women resuscitation efforts or if the condition of the fetus raises serious concerns. - The well-being of the mother should always be a priority. If ibu need stabilization before the act of delivery, this becomes the main thing, as in other obstetric emergencies such as severe preeclampsia and placental abruption. |
|  | Pregnant team members | - Similar to pregnant patients, pregnant health workers should follow infectious disease risk prevention strategies because there is currently no data to support them to stop working, although they may want to consider limiting exposure to high-risk measures, especially in individuals suspected or confirmed to be infected with SARS-CoV- |
|  | Team Training and resource adequacy | - All health facilities providing obstetric care must ensure that their officers are properly trained and able to carry out the recommended infection control interventions, including the use of personal protective equipment. Health workers must ensure that they understand and can comply with infection control requirements. - Adequate equipment and trained health care providers should be available for intrapartum monitoring and obstetric intervention as indicated in separate delivery facilities for infected pregnant women. |
|  | If team members get COVID-19 | - Health workers working in the delivery or neonatal area should report to a supervisor if they have respiratory symptoms or other symptoms that indicate COVID-19 infection. - Such health workers should not be placed on clinical duties and should be replaced by healthy health workers to maintain an appropriate patient-provider ratio.   Health workers who are directly involved in the treatment of patients suspected / proven to be infected with COVID-19 can use hydroxychloroquine (HCQ) prophylaxis |
|  | Protection of Health Workers and wellbeing | Personal Health and Safety   1. The health and safety of all is the paramount priority. Before leaving the lfacility and going home, or before entering the house: wash hands, change clothes and wash with soap and water. 2. Maternity care service personnel need to self-monitor the signs of diseases such as fever, shortness of breath, cough and sore throat, as well as self-isolate and report complaints to managers, if they occur. 3. Workers with COVID-19 symptoms should not work. 4. COVID-19 environment, family, and economic impact-related burnouts, fatigue, and stress can all have an impact on mental and physical health of the workers. It is advisable to manage and seek help if they feel undue signs of stress or have mental health challenges that require supportive intervention. 5. Maternity care workers over the age of 65, those with heart, respiratory, or metabolic conditions, and possibly people with immunodeficiency, need to avoid clinical contact with any patient (not just those suspected of having COVID-19) and consider non-clinical duties if possible. 6. Health care workers with the last trimester of pregnancy or with underlying health conditions such as heart or lung disease at any stage of pregnancy, are advised to avoid direct contact with patients |
